# Unique Characteristics of Women and Infants Moderate the Association between Depression and Mother–Infant Interaction

**DOI:** 10.3390/jcm12175503

**Published:** 2023-08-24

**Authors:** Sandra J. Weiss, Sherryl H. Goodman, Sharon A. Kidd, Margaret Tresch Owen, Diana I. Simeonova, Christine Youngwon Kim, Bruce Cooper, Katherine L. Rosenblum, Maria Muzik

**Affiliations:** 1 Department of Community Health Systems, University of California, San Francisco, CA 94143, USA; bruce.cooper@ucsf.edu; 2 Department of Psychology, Emory University, Atlanta, GA 30322, USA; psysg@emory.edu; 3 Department of Pediatrics, University of California, San Francisco, CA 94143, USA; sharon.kidd@ucsf.edu; 4 Department of Psychology, University of Texas at Dallas, Richardson, TX 75080, USA; mowen@utdallas.edu; 5 Department of Psychiatry and Behavioral Sciences, Emory University, Atlanta, GA 30322, USA; dsimeon@emory.edu; 6 Department of Human Development and Family Studies, Pennsylvania State University, Hershey, PA 17033, USA; ywk1@psu.edu; 7 Departments of Psychiatry and Obstetrics & Gynecology, University of Michigan, Ann Arbor, MI 48109, USA; katier@med.umich.edu (K.L.R.); muzik@med.umich.edu (M.M.)

**Keywords:** depression, postpartum, infants, interactions, women

## Abstract

Research has shown mixed results regarding the association between women’s postpartum depression and mother–infant interactions, suggesting that a woman’s unique experience and context may moderate how depression shapes these interactions. We examined the extent to which a woman’s comorbid anxiety, her exposure to adversity, and infant characteristics moderate the relationship between depressive symptoms of women and interactions with their infants at 6 (*n* = 647) and 12 months (*n* = 346) postpartum. The methods included standardized coding of mother–infant interactions and structural regression modeling. The results at 6 months of infant age indicated that infant male sex and infant negative affectivity were risk factors for mothers’ depression being associated with less optimal interactions. At 12 months of infant age, two moderators appeared to buffer the influence of depression: a woman’s history of trauma and infant preterm birth (≤37 weeks gestation). The results reinforce the salience of infant characteristics in the relationship between maternal depression and mother–infant interactions. The findings also suggest that experiences of trauma may offer opportunities for psychological growth that foster constructive management of depression’s potential effect on mother–infant interactions. Further research is needed to clarify the underlying processes and mechanisms that explain the influence of these moderators. The ultimate goals are to reduce the risk of suboptimal interactions and reinforce healthy dyadic relations.

## 1. Introduction

Evidence continues to emerge that postpartum depression (PPD) in mothers is associated with negative outcomes for infants’ development and health [1,2,3]. Problematic mother–infant interactions have been proposed as a primary mechanism in this association, with many studies supporting this view [4,5,6]. However, some research fails to support an association between PPD in women and troubled interactions with their infants [7,8,9,10]. Collectively, research suggests that parenting quality is multiply determined [11]. The complexity of women’s unique experiences and context may moderate how depression shapes interactions with their infants, resulting in a greater risk of poorer qualities of mother–infant interactions for some dyads than others. In addition, predictive analytics of PPD risk indicate that a multivariate approach is likely essential to explain the complex conditions under which PPD is associated with impaired mother–infant interactions [12,13].

### 1.1. Key Moderators

Consistent with empirically supported models of the determinants of parenting [11], including among mothers with an elevated risk of depression [14], we focused on three key facets of a woman’s context. These include co-morbid anxiety, exposure to adversity, and her infant’s characteristics.

#### 1.1.1. Comorbid Anxiety

Anxiety is the most common comorbidity of women with depression [15,16,17,18]. Their co-occurrence is especially strong postpartum [19,20,21]. Moreover, anxiety has been directly associated with problematic mother–infant interactions [6,22].

#### 1.1.2. Exposure to Adversity

Exposure to adversity can take many forms, but economic hardship and history of trauma are two factors that are strong candidates as moderators of the association between depression and mother–infant interactions. Poverty has been linked to postpartum depressive symptoms [23] and to decreased sensitivity and more negative caregiving [24]. History of trauma is also associated with greater depressive symptoms postpartum, and PPD is exacerbated by varied types of trauma [25,26,27]. Further, post-traumatic stress, exposure to interpersonal violence, and childhood maltreatment are all predictors of more maladaptive interactions with infants [28]. 

#### 1.1.3. Infant Characteristics

##### Infant Sex

The results of two studies examining infant sex and mother–infant interactions indicate mothers’ less positive engagement with daughters during play [15] and more affective matching with daughters [14]. In contrast, two other studies found greater effects of maternal depression on mother–infant interactions for boys than girls [29,30]. Still, other research found no moderating effects of infant sex on the association between mothers’ depression and mother–infant interactions [10,31]. These mixed results support the need for continued study of how infant sex may interact with maternal depression in the prediction of mother–infant interactions, if at all. 

##### Temperament

A systematic review and meta-analysis revealed that mothers’ postnatal depression predicted infant temperament, specifically negative affectivity [32]. The results of another meta-analytic review showed relationships between infant negative affectivity and less sensitive parenting, with a small effect size in general population samples, and a larger effect size in “at risk” samples [33]. Findings that irritability and negative emotionality may render children more susceptible to maternal depression [34,35,36] and negative parenting behaviors [33,37,38] support the need to better understand negative affectivity as a potential moderator of the association between mothers’ PPD and mother–infant interactions.

##### Neonatal Health

Evidence from a recent review shows prevalence rates of PPD increasing to 40% for mothers whose infants are admitted to the NICU [39], along with high levels of mood and anxiety symptoms [40]. Studies also suggest that mothers of infants with more medical complications or who are born preterm (of early gestational age and often low birthweight) may have more intrusive, controlling parenting styles and appear more remote in interactions with their infants than other mothers [41,42,43]. Risks to positive parenting that may stem from neonatal health problems support the need to assess their potential moderating effect on the relationship between postpartum depression and mother–infant interactions. 

### 1.2. Summary

The findings for each of these potential moderators provide a compelling argument for examining their roles in strengthening or reducing associations between mothers’ depression and mother–infant interactions. The field lacks an evidence-based, *multivariate* model of factors that may contribute to suboptimal qualities of mother–infant interactions among women at elevated risk of depressive symptoms. The generation of such a model has been constrained by datasets that are too small to fully test interactions between variables and/or by samples that include women with only one source of risk of depression. 

### 1.3. Study Purpose

To address these gaps, we examined the extent to which a woman’s *comorbid anxiety*, her *exposure to adversity* (poverty and history of trauma), and *infant characteristics* (sex, temperament, and neonatal health) moderate the relationship between mothers’ depressive symptoms and the qualities of mother–infant interactions at infant ages of 6 and 12 months. We expected depressive symptoms to be more strongly associated with less optimal qualities of mother–infant interactions in the context of mothers’ comorbid anxiety, elevated adversity, and infant characteristics that may pose a challenge. Each of these factors may strain the mother’s capacity to interact in optimal ways.

## 2. Method

### 2.1. Design and Procedures

Data and video records of mother–infant interactions were merged from eight previous studies of women with an elevated risk of PPD. Projects were conducted by researchers affiliated with the National Network of Depression Centers who were from three universities in different geographic regions of the U.S. Women in the eight samples were at risk of depression based on their history of major depressive disorder or bipolar disorder, a history of childhood maltreatment, or delivery of a preterm or low-birth-weight infant. Participants were recruited either during pregnancy or in early postpartum at obstetric and/or neonatal units or through advertisements. In regions of the country where Hispanic/Latinx participants were well represented in the population, measures were provided in both English and Spanish. These projects also had members of their research team who were Hispanic and fluent in Spanish. For some projects, English speaking was an inclusion criterion.

Women provided sociodemographic data when recruited and completed depression questionnaires at 6 and 12 months postpartum. We harmonized data from questionnaires across studies to develop common metrics for maternal and infant sociodemographic and clinical characteristics, as well as maternal depression. We used a standardized rating system to analyze the qualities of mother–infant interaction in the video records from all studies. Data were entered into a Research Electronic Data Capture (REDCap) platform at the data coordinating center of the University of California, San Francisco. The Institutional Review Boards of the Human Research Protection Programs at the three universities of participating investigators approved the research projects (Approval code: # 16-19653, 166168). 

### 2.2. Measures

#### 2.2.1. Maternal Depression

The original studies measured the mothers’ depression with 1 of 3 instruments. These included the Beck Depression Inventory (BDI or BDI-II [44,45]), the Postpartum Depression Screening Scale (PDSS [46]), and the Patient Health Questionnaire-9 (PHQ-9 [47]). These measures each have established validity and reliability in perinatal and other populations. We harmonized women’s depression scores by using validated cut-points for depression severity that were determined by the researchers who developed the measures. We grouped women across projects into comparable depression severity groups by using these cut-points (i.e., none or minimal, mild, moderate, and severe). We placed women into unique depression severity groups at both 6 and 12 months postpartum based on their depression scores at these specific points in time.

#### 2.2.2. Mother–Infant Interactions

The video records for all projects provided documentation of mother–infant interactions during an unstructured free-play situation in which mothers were asked to ‘please play with your baby in a way that is typical for you.’ Although the free-play situations differed across studies in whether they were recorded at home or in a behavioral laboratory, we found that neither this difference in setting nor a difference in project site were associated with the maternal interaction scores. In tests of between-subjects effects using a general linear model, the partial eta squared tests for the effect size of the project site ranged from 0.000 to 0.004 (at *p* = 0.34 or greater); η_p_^2^ for the effect size of the setting (lab versus home) ranged from 0.002 to 0.003, at *p* = 0.19 or greater. 

Using a well-established practice (e.g., [48]), we rated the first five minutes of play interaction for each video in order to standardize the segments that were rated across studies. Interactions were rated using a system originally developed for the NICHD Study of Early Child Care and Youth Development [49]. Although these rating scales also included infant rating items and one dyadic item, the parent interaction items were the focus of this research. We also added two items on depressed and anxious mood from the Parent–Child Early Relational Assessment (PCERA [50]) to enhance the salience of the rating system for mood problems common in interactions of women at risk of depression. The resulting measure used for this study consisted of nine scales: *Sensitivity/Responsiveness to Distress, Sensitivity/Responsiveness to Non-Distress, Intrusiveness, Detachment/Disengagement, Stimulation of Development, Positive Regard for the Child, Negative Regard for the Child, Depressed/Withdrawn/Apathetic Mood*, and *Anxious Mood*. Each scale had 5 options, from 1 (not at all characteristic) to 5 (highly characteristic), for the mother’s interaction. There is strong evidence for the reliability and validity of scales in the NICHD rating system, including inter-rater reliability [51], as well as for the PCERA mood scales [52,53,54]. 

Research assistants (RAs) underwent training prior to using the rating scales. They participated in a 2-day workshop with one of the measure’s original authors, including immersion in operational definitions and application of the scales, along with discussion and feedback. They also received training regarding developmental norms and potential differences in maternal interactions with 6- and 12-month-old infants. The RAs then underwent practice sessions and reliability assessments relative to the expert’s “master ratings” of both 6- and 12-month-olds. After training, the RAs were required to achieve reliability on 4 consecutive videos with “master ratings” of an expert who had been responsible for the centralized coding of parent–child interactions in the NICHD study. They were required to differ from the expert by no more than 1 point on no more than 4 scales in their ratings. Once the RAs met this criterion, they independently rated the videos of mothers’ interaction. A second trained observer rated approximately 20% of randomly selected video segments at each infant age to assess inter-rater reliability. Across sites, observers had adequate agreement, as determined by being within 1 point on each rating scale, i.e., greater than 0.80 on all scales. They had agreement between 0.90 and 1.00 on most scales. 

Two final scores for the quality of mother–infant interactions were developed from the ratings for each mother. These scores were derived from exploratory and factor analyses of the data for the 9 rating scales (see [55] for details about the factor analyses). The 2 scores were for the mothers’ *Positive Engagement* with the infant and for her *Negative Intrusiveness*. *Positive Engagement* consisted of five items: positive regard, sensitivity, cognitive stimulation, detachment (negatively loaded), and depressed/withdrawn mood (negatively loaded). *Negative Intrusiveness* consisted of two items: intrusiveness and negative regard. The range for each mother–infant interaction score was from 1 to 5, with one reflecting the lowest level of *Positive Engagement* or *Negative Intrusiveness* and 5 indicating the highest level. We used the means of each of these interaction scores as the final values in analyses. Mothers received separate scores for their interactions with infants at 6 and 12 months of age. 

#### 2.2.3. Moderating Variables

As noted in the aims, three categories of moderators were examined for their potential effects: women’s comorbid anxiety, exposure to adversity, and infant characteristics. 

##### Comorbid Anxiety

The measures of anxiety used across projects included the State-Trait Anxiety Inventory [56], the Generalized Anxiety Disorder Assessment [57], the Anxiety Symptoms Subscale of the Postpartum Depression Screening Scale [46], and the Structured Clinical Interview for DSM-5 [58]. As was the case with our varied measures of depression, we harmonized women’s scores across anxiety measures by using each measure’s validated cut-point for clinically significant symptoms. We then grouped women across projects on a binary score of having clinically significant symptoms of anxiety or not. They received distinct scores for 6 months and 12 months postpartum.

##### Exposure to Adversity

We defined adversity in 2 ways: as poverty and a history of physical or sexual trauma. We determined poverty based on an income level that fell below the federal guidelines for poverty and/or dependence on state/federal services for housing and food [59]. Each of the original studies had assessed these variables through demographic questionnaires collected at enrollment. Based on these data, women were scored as experiencing poverty or not. Across projects, history of trauma was measured with the Childhood Trauma Questionnaire [60], the Psychiatric Diagnostic Screening Questionnaire [61], or the Adverse Childhood Events Questionnaire [62]. Each of the trauma questionnaires had items on physical or sexual abuse (our operational definition of trauma). We assigned a binary score of having experienced trauma or not based upon a woman’s self-report regarding these adverse events in her life. The women received a one-time score for both poverty and trauma. 

##### Infant Characteristics

The original research studies had acquired information on sex and neonatal health through a combination of data from demographic questionnaires and electronic medical records. The neonatal health variables were the infant’s Apgar score, birth weight (BW), and gestational age (GA). For Apgar, we divided the infants into 2 groups as having a score of ≥7 at 5 min after delivery or not. A score of 7 or above indicated good health, with no identified need for extra medical care. BW and GA were continuous variables. Using the GA variable, we also distinguished infants who were born prematurely (<37 weeks gestation) from those who were not. For all the original studies, we measured infant temperament with the very short form of the Infant Behavior Questionnaire–Revised, a well-established caregiver report measure for infants aged 3 to 12 months [63]. The measure yields scores on three broad scales: negative affectivity, positive affectivity/surgency, and regulatory capacity/orienting. Each scale has a possible score ranging from 1 to 7. This measure demonstrates good validity and reliability, including interparent agreement and retest reliability [64]. Because previous research had shown specific evidence for the unique importance of infant negative affectivity, we used this temperament score in this study. The infants received a score for negative affectivity at 6 and again at 12 months of age.

### 2.3. Data Analysis

We calculated descriptive statistics to characterize the sample and correlations to examine preliminary relationships among study variables. To test the contribution of each individual potential moderator, we initially computed separate regressions for each moderating variable, whereby the 2 mother–infant interaction scores were regressed on depression, the potential moderator, and the interaction between depression and the moderator at both the 6- and 12-month time points. In addition to the assessment of our moderator candidates, we examined maternal age, education, and marital/partner status as potential covariates that might need to be controlled for in the final analyses. To test our proposed multivariate model, all covariates, moderators, or interactions that demonstrated a *p* = 0.10 or better level of significance were included in a final structural equation model to assess moderating effects. The use of Full Information Maximum Likelihood (FIML) with the Expectation–Maximization algorithm allowed all participants to be included in the analysis, with data missing at random. We carried out statistical analyses with Stata 16 and Mplus, evaluation tests of significance with a two-sided alpha of 0.05.

## 3. Results

### 3.1. Participant Characteristics

In Table 1, the participants’ characteristics are described for both the mother and infant. This study included 647 mothers and infants at 6 months and 346 mother–infant dyads at 12 months of infant age. We had a smaller sample at the 12-month point because two of the eight projects included in the study had not collected 12-month data. At recruitment, the average age of women was 31.3 years old (±5.9). Approximately 66% were White/European American, 15% were Black/African American, 10% were Hispanic American/Latina, and 6% were Asian American; 63% of the women had a bachelor’s degree or some graduate education; 7% were below the poverty level and receiving government assistance; and 87.5% were married or lived with a partner. 

At 6 months postpartum, 22.4% (*n* = 145) of the women reported moderate to severe depressive symptoms, while the remainder (77.6%) had mild or minimal symptoms; 13.6% (*n* = 47) of the women were moderately to severely depressed at 12 months postpartum, with 86.4% having mild or minimal symptoms. 

The infants’ (50.1% female) mean gestational age at birth was 36.7 weeks (±4.1), with 9.4% having an Apgar score indicating medical concern at birth (<7). On average, the infants were at the middle of the range for scores on negative affectivity at 6 months of age (M = 3.66 out of 7 possible) and at 12 months (M = 4.17); both scores were out of 7 possible. For mother–infant interactions, the average scores for positive engagement were 3.58 at 6 months and 3.56 at 12 months (both scores out of a possible 5). The average scores for negative intrusiveness were 1.50 at 6 months and 1.34 at 12 months (both out of a possible 5).

### 3.2. Bivariate Correlations

Table 2 presents the correlation coefficients for the relationships between the study variables and women’s depressive symptoms at both 6 and 12 months postpartum. Of the variables we selected as potential moderators, women’s anxiety and exposure to adversity showed direct associations with depression at one time point or both. All the neonatal health and infant temperament characteristics were significantly associated with depression at one time point or another. Sex was the only infant characteristic showing no direct association with women’s depression. For covariates, a younger age of women was associated with more severe depression, as were a lack of a partner and less educational preparation. 

The correlations of the study variables with mother–infant interactions are shown in Table 3. Depression was significantly associated with more *negative intrusiveness* of women with their infants at both 6 and 12 months of infant age. Among the variables we examined as potential moderators, women’s anxiety, poverty, and history of trauma, as well as infant sex, gestational age, and negative affectivity, were all directly associated with either *positive engagement* or *negative intrusiveness* of women with their 6- or 12-month-old infants at one or both ages. For the covariates, women’s older age, a higher number of years of education, and the presence of a partner were all associated with more optimal mother–infant interactions. 

### 3.3. Moderators of the Relationship between Women’s Depression and Their Interactions 

#### 3.3.1. Positive Engagement at Six Months of Infant Age

**Figure 1:**
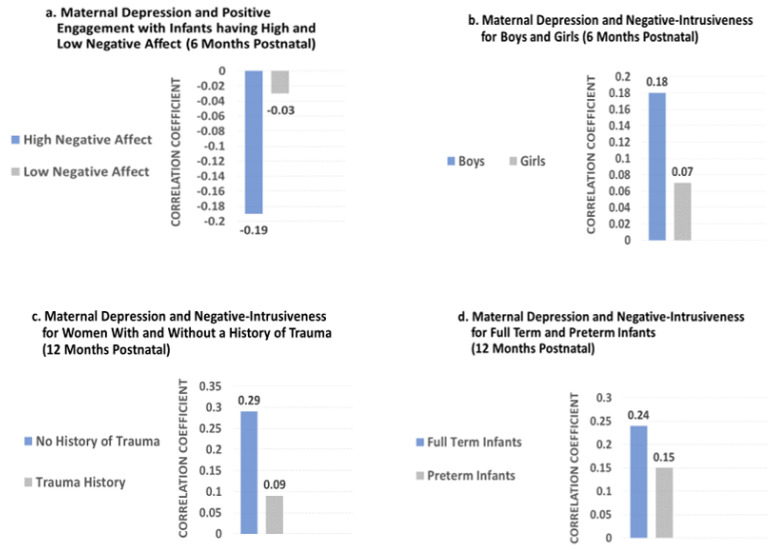
Correlation Coefficients for the Relationship of Maternal Depression with Women’s Interactions with Their Infants among Significant Moderator Groups.

Table 4 provides the results of the final regression model at 6 months of infant age, including all moderators and covariates for which we found a significant effect in preliminary model testing. Infant negative affect moderated the effects of depression on *Positive Engagement*, *B* = −0.100, *p* = 0.039. Separate regression models for infants with high and low negative affect scores showed that maternal depression was associated with lower *Positive Engagement* among dyads with infants who were high in negative affect (*B* = −0.19, *p* = 0.017), but depression was not related to *Positive Engagement* in dyads with infants who were low in negative affect, *B* = −0.03, *p* = 0.54; Figure 1a. In addition to these moderating effects, two candidate moderators had direct effects on *Positive Engagement.* Mothers who met the poverty threshold exhibited less *Positive Engagement* with the infant (*B* = −0.500, *p* = 0.001) and daughters experienced less *Positive Engagement* from their mothers than sons, *B* = −0.182, *p* = 0.028. One of the covariates also had a direct association: mothers with a higher number of years of education showed more *Positive Engagement*, *B* = 0.135, *p* = 0.001. Maternal depression was not directly associated with *Positive Engagement*.

#### 3.3.2. Negative Intrusiveness at 6 Months of Infant Age

More severe maternal depression significantly predicted a mother’s *Negative Intrusiveness* toward the infant at the first step of the regression model, *B* = 0.207, *p* = 0.002. However, the interaction term for depression and infant sex (Table 4) indicated that the effect of maternal depression on *Negative Intrusiveness* was moderated by infant sex, *B =* 0.233, *p* = 0.047. The examination of separate regression models for boys and girls showed that there was no effect of maternal depression on *Negative Intrusiveness* for girls (β = 0.07, *p* = 0.19), but a significant effect for boys (β = 0.18, *p* < 0.001; Figure 1b). Two candidate moderators had direct effects in the model. Poverty and infant sex were associated with mothers’ *Negative Intrusiveness*. Mothers who experienced poverty had a greater probability of interacting with their infants in intrusive, negative ways, *B* = 1.022, *p* = 0.000. In addition, daughters experienced more *Negative Intrusiveness* from their mothers, *B =* 0.216, *p* = 0.044. 

In preliminary model testing, the results indicated that, for women who did not experience trauma, more severe depression was associated with greater negative intrusiveness in their mother–infant interactions (*B* = 0.13, *p* = 0.02). In contrast, for women who did experience trauma, more severe depression was not associated with greater negative intrusiveness (*B* = 0.07, *p* = 0.41). However, history of trauma was not retained in the final model at 6 months of age. As noted above, other variables assumed more salience in predicting negative intrusiveness (i.e., poverty and infant sex).

#### 3.3.3. Mother–Infant Interactions at 12 Months of Infant Age

While no variables significantly moderated the relationship between depression and *Positive Engagement*, two variables did moderate the strength of the association between depression and *Negative Intrusiveness*: a history of trauma (*B* = −0.662, *p* = 0.013) and infant gestational age (*B =* 0.136, *p* = 0.009; Table 5). For women with a history of trauma, there was no significant association between their depression and the probability of *Negative Intrusiveness* with their infants, *B* = 0.08, *p* = 0.50. However, for women without any history of trauma, more severe depression increased the probability of them using *Negative Intrusiveness* (*B* = 0.28, *p* = 0.001, Figure 1c). 

If infants were of greater gestational age, their mothers’ depression was more strongly associated with *Negative Intrusiveness.* To better understand this moderating effect, we examined differences in this relationship for a clinically meaningful cutoff specific to gestational age: <37 weeks gestation (born prematurely) versus 37 weeks gestation or later (full-term birth). For infants born prematurely, their mothers’ depression was not related to negative intrusive interactions, *B* = 0.14, *p* = 0.33. However, for infants born full term, depressive symptoms of mothers were significantly associated with more negative intrusive behaviors when interacting with their infants (*B* = 0.19, *p* = 0.003; Figure 1d). 

The covariate of maternal age had a significant relationship with both types of mother–infant interactions. Older mothers showed more *Positive Engagement* with their infants, *B =* 0.044, *p* = 0.000. In contrast, younger mothers used more *Negative Intrusiveness* with infants, *B* = −0.078, *p* = 0.000. Maternal depression was not associated with either mothers’ *Positive Engagement* or *Negative Intrusiveness* at 12 months of infant age.

## 4. Discussion

Among our sample of women with different risk factors for PPD, we found that 41.6% of women had at least mild depressive symptom levels at six months and 28.6% at twelve months postpartum. This finding is consistent with those of other studies on postpartum women with risk factors, such as prior episodes of major depression [65]. Thus, our sampling strategy was effective in generating a sample of women with elevated depressive symptom levels relative to the general population. 

We found support for four of the six candidate variables as moderators of the relationship between PPD and mother–infant interactions. Depressive symptoms were more strongly associated with mother–infant interactions when infants were male, had temperaments with higher levels of negative affectivity, and were of greater gestational age. Depression was also more strongly linked to mother–infant interactions in the context of mothers not having a history of trauma. The findings differed by the infants’ age at the time of the observed interaction. At 6 months of age, mothers’ depression was more strongly associated with reduced positive engagement among infants with higher negative affectivity and with greater *negative intrusiveness* with sons rather than with daughters. At 12 months, mothers’ depression was more strongly associated with negative intrusive interactions among women without any history of trauma (compared with women who had experienced trauma) and among infants with greater gestational age (including full-term compared with preterm infants). 

Comorbid anxiety and poverty did not significantly moderate the association between PPD and mother–infant interactions. However, poverty had a direct association with mother–infant interactions, increasing the risk of mothers’ *negative intrusiveness* with infants. In addition, two covariates were associated with mother–infant interactions. Mothers who were older and mothers who had a higher number of years of education showed greater *positive engagement* with their infants. 

Our findings indicate that different factors are moderating the association between depression and mothers’ interactions at different stages of infant development; that is, 6 and 12 months of infant age. It may be that more immediate contexts involved in getting to know one’s baby, such as the baby’s negative affectivity and sex, influence the depression–parenting relationship more at younger infant ages (6 months of age), whereas contextual stressors, such as history of abuse or caring for a medically fragile preterm infant, have a stronger influence later in the postnatal period (12 months of infant age). Planalp et al. [66] similarly found differential prediction of parenting qualities at different infant ages. Future research might include more age points to examine the developmental trends in the role of various moderators. If our findings are replicated, they suggest the importance of taking a developmental perspective in addressing factors that may moderate relationships between mothers’ depression and their interactions with infants.

### 4.1. Significant Moderators

Three of the four variables for which we found a significant moderating effect were infant characteristics, suggesting the salience of this category of moderators in the association between maternal depression and mother–infant interactions. These infant characteristics may each contribute to a dyadic context that either calms or perturbs the underlying depressive symptoms experienced by a mother, supporting or diminishing her ability to keep her depression from influencing her interactions with the infant. 

#### 4.1.1. Infant Sex

We found that more severe depressive symptoms were associated with more *negative intrusiveness* to a greater extent with sons than with daughters. Although some studies have found no moderating effect of infant sex [10,31], our finding is consistent with others who have reported greater vulnerability for boys than girls to the negative effects of maternal postpartum depression on mother–infant interactions (e.g., [29,30]). Specifically, Weinberg et al. [30] found that more negative affectivity was displayed among more severely depressed mothers in Still-Face-Paradigm reunions with their 6-month-old sons than with their daughters. The findings reported by Tronick and Reck [29] for mother–infant interactions observed in the lab and in the home highlighted greater emotional reactivity among sons than daughters of mothers with elevated depressive symptoms, which could suggest that boys present particular challenges for mothers with more severe depressive symptoms. 

#### 4.1.2. Infant Temperament

Among infants with more negative affectivity, we found that mothers’ depression was strongly related to less *positive engagement* with the infants. This moderating effect is consistent with earlier findings that negative affectivity can put infants at greater risk from maternal depression and less optimal parenting [34,36,38,67]. Newland et al. [68] specifically reported a moderating effect akin to our finding, whereby maternal depression was only associated with reduced sensitivity among infants who displayed heightened negativity during interactions. Thus, our results provide additional support for the view that less infant negativity might serve to buffer the effects of maternal depression, while greater negativity may intensify its effects in mother–infant interactions. Mothers may experience stress and difficulty in interacting sensitively with an infant who displays frequent negative affect and related trouble being soothed, especially mothers experiencing elevated symptoms of depression.

#### 4.1.3. Neonatal Health

Of the three neonatal health variables we examined (gestational age (GA), Apgar score, and birthweight), only GA moderated the association between depression and mothers’ interactions with infants. Although we found that more severe depressive symptoms were related to more negative intrusive interactions with the infant at 12 months of age, this relationship was only observed for infants who were of older gestational age at birth and born full term. Our findings suggest that the mothers of preterm infants may attempt to regulate their depressed mood state so that it is not associated with negative intrusive interactions with the infant. In fact, Agostini et al. [69] reported that mothers of preterm infants were engaged with their infants in positive ways, even when depressed. Research indicates that mothers of preterm and other medically fragile infants express strong concerns about providing too much stimulation to the infants and whether their caretaking could place the infants at greater medical risk [70,71]. Such concerns would not be present for full-term, more developmentally mature infants, allowing for less regulation of a mother’s negative emotion and depression’s influence on mother–infant interactions. 

#### 4.1.4. History of Trauma

A woman’s history of trauma was the only adversity variable we examined that showed a moderating effect. While depression was associated with a woman’s negative intrusiveness during interactions with her infant if she had no history of trauma, a woman’s previous experience with trauma appeared to buffer this adverse effect of depression on her interactions with the infant. Although this finding only emerged in the final model at 12 months postpartum, we did see the same result in the preliminary phases of model testing at 6 months postpartum. This finding clearly was unexpected and counterintuitive. However, previous theories and research may shed light on a potential rationale for our results. It has long been recognized that parents with a history of trauma may experience ‘triggering’ of trauma responses when they are caring for their infant, especially when the infant is experiencing distress [72]. However, it has been hypothesized in ‘inoculation theory’ that individuals with a history of prior stress may be able to use their past experience to better cope with later traumatic events and be more resistant to subsequent stressors [73,74]. Some research indicates that stress inoculation enhances prefrontal myelination and cortical expansion induced by the process of coping, and results in enduring trait-like transformations in cognitive, motivational, and emotional aspects of behavior [75,76]. Research on ‘Posttraumatic Growth’ (PTG) has built upon this theory, as it relates specifically to individuals with a trauma history. PTG refers to a process of cognitive adaptation and positive psychological change that can emerge following exposure to trauma, which may be the result of having survived the trauma and seeing new possibilities in life [77]. It has been associated with increased introspection, a more realistic appraisal of potential difficulties, enhanced interpersonal functioning, and less reactivity to potentially unsettling PTSD symptoms, among other improvements [78,79]. Although we have no direct evidence from our study to support the hypothesis, women in our study who had a trauma history may have experienced stress inoculation and related posttraumatic growth, improving their psychological ability to reflect on their depression and its potential impact on their mother–infant interactions. It is also possible that women with a history of trauma may have engaged in psychotherapy and strengthened their posttraumatic growth through treatment. As noted earlier, we have no evidence to support these conjectures. They will require confirmation in future research before assuming their validity.

### 4.2. Candidate Moderators That Were Not Significant

#### 4.2.1. Anxiety

Although preliminary correlations suggested relationships of maternal anxiety with both depression and mother–infant interactions, these relationships did not persist after adjusting for other variables in the regression models. In our final models, maternal anxiety had neither a moderating effect on the relationship between depression and maternal interaction, nor a direct relationship with the quality of interactions with infants. Although there has been little support for the moderating effect of anxiety in previous studies, research has shown an association between maternal anxiety postpartum and both less sensitivity [80] and greater intrusiveness [81] toward infants. However, in contrast with our measurement of mothers’ currently experienced anxiety symptoms, those earlier studies examined the effects of trait anxiety or mixed trait–state assessments combined into a single measure of anxiety. Congruent with our findings, research assessing the effects of current anxiety disorders/symptoms has found no differences in maternal sensitivity or intrusiveness between women experiencing anxiety versus those who did not (e.g., [6,82]). Research is needed to differentiate the potential influence of an anxious, more persistent predisposition versus the acute experience of anxiety on mothers’ interactions with their infants.

#### 4.2.2. Poverty

Although it had no moderating effect, poverty was a significant predictor of both less *Positive Engagement* and greater *Negative Intrusiveness* at 6 months of infant age. There is evidence from previous research that mothers with lower income show less sensitivity and less growth-fostering behavior with their infants [83]. A recent study also found that the probability of using less optimal parenting behaviors increased incrementally with lower incomes [84]. In conjunction with these studies, our findings imply that poverty has a direct, negative effect on mother–infant interactions, rather than influencing how depression affects a mother’s interactions. The stress and added challenges of poverty may leave women with few emotional resources to attend effectively to their infant’s needs [23]. However, the influence of poverty on mothers’ interactions did not persist to 12 months of infant age, suggesting that other factors become more influential over time.

### 4.3. Covariates: Maternal Age and Education

Of the three covariates we included to control for their effects, maternal education and age were significantly associated with mother–infant interactions. A higher education level was associated with more *Positive Engagement* with the infant at 6 but not 12 months of age, while older age of mothers predicted both more *Positive Engagement* and less *Negative Intrusiveness* at 12 months of infant age. These results are consistent with other research that has shown evidence of more highly educated mothers interacting with their infants in more positive, developmentally appropriate ways [85,86]. Higher education has been associated with a better ability to read infant cues [87], as well as more positive affect and less intrusive behavior [88]. Similarly, evidence indicates that older mothers are more sensitive and responsive to their infants [89] and have enhanced child-rearing skills [90]. As Bornstein et al. [91] noted, the link between maternal age and positive parenting is linear. The less positive parenting observed among mothers of younger age and lower education may be attributed to their limited knowledge of child development and increased parenting stress [90,92]. 

### 4.4. Limitations and Strengths

The limitations of the study are important to note. Most women had more than a high school education, only one-fifth were in poverty and most were living with a partner, reducing generalizability to women and infants who may have less security in their lives. Certain groups of women at elevated risk of depression were not well-represented in our sample, including immigrants who spoke languages other than English or Spanish. In addition, the sample was not well-distributed racially, with no representation of Indigenous, American Indian, or Inuit women. The measures used may have been validated with predominantly White populations and have less or unknown validity among women from other racial or ethnic groups.

Despite the robust sample size, a few of our moderating variables did not have as strong a distribution as hoped, decreasing the power to detect their effects (e.g., poverty). Two of the projects from which the data were leveraged did not collect data at 12 months of infant age, resulting in a smaller sample size for analysis at that time. Harmonizing data from previously conducted studies involved an intensive review of available measures and their potential comparability across projects. A few of the questionnaires we used to provide data on similar constructs had slightly different conceptualizations of the construct. More broadly, we were limited by the data that had previously been collected in terms of the moderators we could examine.

However, this study had many strengths. While previous studies have each focused on one group of women who were at risk of PPD (e.g., women with a history of depression), our sample represented a group of women with varied factors that elevated their risk of PPD (a history of major depressive disorder or bipolar disorder, a history of childhood maltreatment, and delivery of a preterm or low birth weight infant). This broader representation enhances the generalizability of the findings across a spectrum of women who are at risk of depression. The women were also from distinctly different regions of the U.S. In addition, we moved beyond main effects models to examine moderating effects when examining the relationship between women’s depression and mother–infant interactions. Our hypothesized moderators were theory- and evidence-driven. In contrast with most studies of moderation with small samples, we combined data across eight studies to enhance the power available for testing moderating effects. We examined mother–infant interactions at two developmental time points using an established, reliable measure that we applied to the video-recorded interactions of all the studies. Lastly, we applied integrative statistical techniques to create common metrics across studies and harmonize uniquely different datasets to leverage existing data. These strengths supported the development of new knowledge beyond that provided by previous studies. 

### 4.5. Implications for Research and Practice

Our results contribute to an improved understanding of the moderating context (i.e., for whom or under what circumstances) within which the relationship between depression and mother–infant interactions may be stronger or weaker. The findings support a focus on moderating factors as a fruitful area for continued research. The ways in which infant sex, temperament, and gestational age interact with one another as potential moderators may be especially important to examine, requiring an even larger sample than ours. In addition, our findings regarding trauma warrant further study to better understand how a woman’s history of trauma may enhance her ability to modulate any effects of depression on interactions with her infant. For instance, the potential influence of the increased empathy or insight developed by women who experience trauma could be examined. Another focus of needed research is to evaluate differences in the potential moderating effects of a woman’s general predisposition to anxiety versus her experience of acute anxiety symptoms on the ways in which her depression may influence interactions with her child. Lastly, different areas of financial insecurity should be examined for their potentially unique roles as moderators. Our general measure of poverty may have missed important nuances associated with this variable.

Further research in these areas can eventually enable the targeting of interventions to key moderators to reduce the infant’s risk of later mental health problems that have been associated with maternal depression. Given limited resources, the moderators also provide guidance on the prioritization of services. Ultimately, improving mother–infant interactions among the highest-risk groups can reduce the growing incidence of childhood psychopathology and contribute to a healthier society in the long term. Understanding key moderators, such as the infant characteristics identified in our research, can improve clinicians’ ability to tailor interventions to subsets of women and infants for whom depression may have the strongest potential to be associated with problems in their interaction. Our research has I generated foundational knowledge to enhance this goal. 

## Figures and Tables

**Table 1:** Maternal and Infant Characteristics.

Variables		
**Maternal Sociodemographic Characteristics**	**6 Months**	**12 Months**
	**M (SD)**	**M (SD)**
**Maternal Age**	31.3 (5.9)	32.2 (5.2)
**Highest Level of Education**	**N (%)**	**N (%)**
Less than High School (HS)	50 (7.7)	5 (1.4)
HS Graduate or GED	49 (7.5)	19 (5.3)
Some College/Vocational/Other Post-HS	136 (21.1)	73 (20.3)
Bachelor’s Degree	195 (30.1)	125 (34.8)
Post-Graduate	217 (33.6)	137 (38.2)
**Race/Ethnicity**		
White/European American	427 (66.1)	272 (75.6)
Black/African American	97 (15.0)	45 (12.5)
Hispanic/Latina	64 (9.8)	14 (3.9)
Asian Americanth	37 (5.7)	20 (5.8)
More than One Race	22 (3.4)	9 (2.5)
**Partner Status**		
Married/Living with Partner	566 (87.5)	337 (93.4)
Single/Separated/Divorced/Widowed	81 (12.5)	24 (6.6)
**Poverty Level**		
Below Poverty Level	112 (17.3)	33 (9.2)
**Maternal Clinical Status and History**	**6 Months**	**12 Months**
**Depression**	**N (%)**	**N (%)**
Minimal/No Depression	378 (58.4)	247 (71.4)
Mild	124 (19.2)	52 (15.0)
Moderate	95 (14.7)	27 (7.8)
Severe	50 (7.7)	20 (5.8)
**Met Threshold for Clinical Anxiety**	188 (29.1)	97 (28.0)
**History of Physical or Sexual Abuse**	212 (32.9)	108 (31.5)
**Infant Characteristics**	**6 Months**	**12 Months**
**Sex**	**N (%)**	**N (%)**
Male	323 (49.9)	182 (49.1)
Female	324 (50.1)	189 (50.9)
**Apgar Score < 7**	61 (9.4)	7 (2.4)
	**M (SD)**	**M (SD)**
**Birthweight in Grams**	2973.1 (952.7)	3290.1 (580.2)
**Gestational Age in Weeks**	36.7 (4.1)	37.7 (3.4)
**Temperament (Negative Affectivity)**	3.66 (0.90)	4.17 (0.92)
**Mother–Infant Interaction**		
Positive Engagement	3.58 (0.61)	3.56 (0.60)
Negative Intrusiveness	1.50 (0.68)	1.34 (0.52)

**Table 2:** Correlations of Potential Moderators and Covariates with Women’s Depression at 6 and 12 Months Postpartum (PP).

Moderators	Depression at 6 Months PP	Depression at 12 Months PP
**Woman’s Anxiety**		
6 Months PP	0.32 **	0.23 **
12 Months PP	0.16 **	0.29 **
**Exposure to Adversity**		
Poverty	0.19 **	0.26 **
Trauma	0.09	0.24 **
**Infant Characteristics**		
Sex (M)	0.01	−0.04
Apgar < 7	0.26 **	−0.07
Birthweight	−0.25 **	0.02
Gestational Age	−0.23 **	0.07
**Negative Affectivity**		
6 Months PP	0.17 **	0.18 **
12 Months PP	0.06	0.18 **
**Covariates**		
**Woman’s Age**	−0.24 **	−0.13
**No Partner**	0.23 **	−0.27 **
**Woman’s Education**	−0.29 **	−0.19

** *p* ≤ 0.001.

**Table 3:** Correlations between Study Variables and Women’s Interaction with Their Infants at 6 and 12 Months of Infant Age.

	6 Months Postnatal		12 Months Postnatal	
	Positive Engagement	Negative Intrusiveness	Positive Engagement	Negative Intrusiveness
**Depression**	−0.08	0.18 **	0.05	0.12 **
**Moderators**				
**Woman’s Anxiety**				
6 Months PN	−0.12 *	0.03	−0.30 **	0.12 *
12 Months PN	−0.11 **	−0.02	−0.06	0.07
**Exposure to Adversity**				
Poverty	0.19 **	0.30 **	−0.09	0.21 **
Trauma	−0.03	0.11 *	−0.01	0.08
**Infant Characteristics**				
Sex (M)	−0.07	0.05	0.12 *	−0.03
Apgar < 7	−0.01	0.02	0.01	−0.07
Birthweight	0.01	−0.06	0.02	−0.07
Gestational Age	−0.04	−0.03	−0.13 *	0.02
**Negative Affectivity**				
6 Months PN	−0.10 *	0.08	−0.13 *	0.12 *
12 Months PN	−0.05	0.14 *	−0.06	0.05
**Covariates**				
Woman’s Age	0.15 *	−0.19 **	−0.19 **	−0.25 **
No Partner	−0.16 *	0.20 **	−0.11 *	0.23 **
Years of Education	0.22 **	−0.23 **	−0.17 **	−0.21 **

* *p* ≤ 0.01; ** *p* ≤ 0.001.

**Table 4:** Effects of Depression and Significant Moderators on Mothers’ Positive Engagement and Negative Intrusiveness with their Infants at 6 Months of Age.

	Coefficient	SE	*p*-Value	95% Confidence Intervals
**Positive Engagement** **Mother**				
Depression Severity	−0.034	0.05	0.530	−0.138, 0.071
Education Level	0.135	0.04	0.001	0.054, 0.217
Poverty	−0.500	0.15	0.001	−0.803, −0.198
**Infant**				
Sex	−0.182	0.08	0.028	−0.345, −0.020
Gestational Age	−0.021	0.01	0.058	−0.043, 0.001
Negative Affectivity	−0.089	0.05	0.070	−0.184, 0.007
**Interactions**				
Depression × Negative Affectivity	−0.100	0.05	0.039	−0.195, −0.005
**Negative Intrusiveness Mother**				
Depression Severity	0.207	0.07	0.002	0.075, 0.340
Poverty	1.022	0.19	0.000	0.653, 1.390
**Infant**				
Sex	0.216	0.11	0.044	0.006, 0.426
**Interactions**				
Depression × Sex	0.233	0.12	0.047	0.003, 0.462

**Table 5:** Effects of Depression and Significant Moderators on Mothers’ Positive Engagement and Negative Intrusiveness with their Infants at 12 Months of Age.

	Coefficient	SE	*p*-Value	95% Confidence Intervals
**Positive Engagement** **Mother**				
Depression Severity	0.042	0.06	0.523	−0.087, 0.171
Age	0.044	0.01	0.000	0.020, 0.067
**Negative Intrusiveness** **Mother**				
Depression Severity	0.042	0.13	0.759	−0.310, 0.226
Age	−0.078	0.02	0.000	−0.115, −0.041
History of Trauma	0.045	0.21	0.831	−0.365, 0.454
**Infant**				
Gestational Age	0.016	0.02	0.522	−0.034, 0.067
**Interactions**				
Depression × Trauma	−0.662	0.26	0.013	−1.184, −0.141
Depression × GA	0.136	0.05	0.009	0.003, 0.239

## Data Availability

The data underlying the results presented in this study are available on request from the corresponding author.
